# Analysis of the monitoring of the expansion of Covid-19 testing and surveillance in a municipality

**DOI:** 10.11606/s1518-8787.2026060006945

**Published:** 2026-05-01

**Authors:** Alcione Brasileiro Oliveira, Ana Maria Freire de Souza Lima, Thais Regis Aranha Rossi, Gerluce Alves Pontes da Silva, Sisse Figueiredo de Santana, Trícia Silva Santos, Izabel Cristina Neves Ramos, Sintique Priscila Alves Lopes, Nathalia Almeida Suzart, Laio Magno, Inês Dourado, Lígia Maria Vieira da Silva

**Affiliations:** I Universidade Federal da Bahia. Instituto de Saúde Coletiva. Salvador, BA, Brasil; II Universidade Federal da Bahia. Faculdade de Odontologia. Departamento de Odontologia Social e Pediátrica. Salvador, BA, Brasil; III Universidade do Estado da Bahia. Departamento de Ciências da Vida. Salvador, BA, Brasil; IV Secretaria Municipal de Saúde de Camaçari. Camaçari, BA, Brasil; V Fundação Oswaldo Cruz. Instituto Gonçalo Moniz. Salvador, Bahia, Brasil

**Keywords:** Sanitary Supervision, COVID-19, Primary Health Care, Digital Health Technology, COVID-19 Testing

## Abstract

**OBJECTIVE:**

To analyze the monitoring of an intervention for expanding testing, isolation, quarantine, and telemonitoring of Covid-19 (TQT-Covid-Strategy) in an administrative health region of a municipality in Northeastern Brazil.

**METHODS:**

This is an evaluative study, whose object of analysis were data produced in the monitoring of a health intervention (TQT-Covid-Strategy), for six months, in 17 health units, namely 12 Family Health Units and five Health Centers. Monitoring matrices created through field reports, workshops with professionals and managers, and permanent education activities were analyzed. Monitoring took place in the three components of the TQT-Covid-Strategy intervention: expansion of accessibility to testing, monitoring of cases and surveillance strategies, and digital platform. The actions in each component were considered adequate (A), partially adequate (P), and inadequate (I) in relation to the activities determined in the action plan and in the protocol of the TQT-Covid-Strategy.

**RESULTS:**

The component of the expansion of accessibility to testing was considered adequate, while the monitoring of cases and surveillance strategies presented partially adequate or inadequate results in many units. As for the digital platform component, there was predominantly adequate performance in relation to registration and access to test results and case reporting. However, the use of other surveillance-related resources, such as contact tracing, was inadequate.

**CONCLUSIONS:**

Boosting the institutionalization of monitoring can be an important instrument for the implementation and improvement of health interventions. The regular presence of enablers and a widely disseminated protocol, in addition to community health agents, enhanced the intervention. However, partially adequate or inadequate results reinforced the importance of qualification of the work process in primary health care regarding surveillance actions and the use of information and communication technologies.

## INTRODUCTION

The health emergency declared by the World Health Organization in February 2020, followed by the confirmation of the Covid-19 pandemic, caused over 770 million cases and almost 7 million deaths by 2023^1^. The situation led countries to adopt several measures, the main ones being testing, identification of cases and their isolation, contact tracing, and quarantine, central components of epidemiological surveillance^2–4^, in addition to the reorganization of care, generating harmful consequences, especially for those with greater social inequality^2,4^.

Primary Health Care (PHC) was paramount in the expansion of testing and other coping strategies for Covid-19 worldwide^5–7^. Countries, such as Canada, the United Kingdom, Portugal, Spain, and New Zealand, also stood out by rapidly implementing monitoring and telemonitoring systems through PHC to support patients and engage in the monitoring of chronic and acute diseases and telemedicine tools^8–11^.

In Brazil, although health centers (*unidades básicas de saúde* – UBSs) and Family Health Units (FHUs) are present in all municipalities, including the most remote areas of the country, there is little evidence about the monitoring of the interventions proposed during the pandemic, the incorporation of new systems, resources, and surveillance actions by PHC teams as well as the sustainability of this incorporation and knowledge for future scenarios^12–14^. In particular, regarding the use of information and communication technologies, limitations for implementation are highlighted, especially in places with greater socioeconomic vulnerability, with more difficulty in accessing the Internet, and low digital literacy^15,16^.

In a municipality in the Northeast of Brazil, a strategy was implemented for expanding testing, isolation, quarantine, and telemonitoring of Covid-19 (TQT-Covid-Strategy) in 17 PHC health units (HUs) in an administrative health region (AHR)^17,18^. It should be noted that the implementation of new intervention must be monitored and evaluated through a formative approach, as it enables to identify, at an early stage, if the expected results are being achieved, make corrections, in addition to increasing the probability of obtaining reliable summative results at the end of the process^19^.

In the present study, we analyzed the monitoring of this intervention, the TQT-Covid-Strategy, for six months, with weekly observation of HUs. The results can contribute to preparations for facing future pandemics, through accumulated experiences, knowledge, and legacies.

## METHODS

This is an evaluative study, whose object of analysis were data produced while monitoring the TQT-Covid-Strategy, from July 2022 to February 2023, in 17 HUs of an AHR of a municipality in Northeastern Brazil, being 12 FHUs and five UBSs. The monitoring took place for six months, with observations conducted by a team of researchers and weekly visits to HUs. The monitoring was understood as an evaluation that takes place over time^20^.

The formative approach was adopted for the assessment, using the classification proposal adapted by Stetler et al.^19^ Monitoring involved the stages focused on implementation, i.e., on the process, by field monitoring, according to the intervention plan, and on progress, seeking to achieve the implementation goals, identifying aspects to be addressed through feedback for teams and refinement of strategies^19^.

### Analysis of the Initial Situation

The municipality where the analyzed AHR is located is home to approximately 3 million inhabitants and 483 HUs, corresponding to a PHC population coverage of 57.68%^20^. The AHR where the intervention took place covers a population of almost 400 thousand inhabitants, with 22 PHC units, of which 10 UBSs and 12 FHUs^21^.

The design of the TQT-Covid-Strategy was developed through situational analysis and diagnosis of the main difficulties for coping with the pandemic in the aforementioned AHR. Initially, four meetings were held in December 2021, with 33 participants (managers, health professionals, and researchers). The second phase of this evaluation followed until March 2022, with semi-structured interviews (professionals and users) and focus groups (professionals)^22^. The main problems identified before the intervention were related to infrastructure (materials, inputs, and human resources), organization and management as well as the work process (Chart 1).

**Chart 1:** Main issues identified in the administrative health region of a municipality in Northeastern Brazil prior to the intervention (2021–2022).

Infrastructure, materials, inputs
Inadequate infrastructure
Lack of electronic devices and unstable Internet connection
Lack of inventory control of PPE and inputs for testing
Human resources
Lack of professionals
Work overload and workers’ illnesses
Organization and management
Testing concentrated in few health units
Lack of preparation and uncertainties about protocols and flows and lack of provision of training on Covid-19 by municipal management
Lack of communication channel with users
Manual completion of notification forms and notification problems in the Ministry of Health system
Lack of disclosure of the occurrence of Covid-19 cases disaggregated by coverage area of health units
Monitoring of the situation manually and/or using Microsoft Excel and Google spreadsheets, with discontinuities
Work process
Low testing with presence of lines and long waiting time in health units
Lack of health communication strategies in the territories on Covid-19 (prevention, symptoms, testing, and vaccination)
Lack of early identification of cases and contacts
Low case notification
Low active search index (new cases and contacts)
Nonexistent or poor telemonitoring
Difficulties in carrying out, recording, and systematizing telemonitoring — most of them done manually
Difficulty accessing the test reports
No contact tracing

PPE: personal protective equipment.

Source: Formative Assessment Report of the TQT-Covid-Strategy.

Of the 22 HUs, five were excluded because they were managed by the private sector or were without nursing professionals and/or with very limited physical structure. Thus, the TQT-Covid-Strategy involved 17 HUs, 12 FHUs and five UBSs. It should be noted that, of the 17 HUs, seven already carried out tests for Covid-19 or had some experience with this activity; the others began to perform tests after implementing the TQT-Covid-Strategy; three FHUs were implemented in the pandemic period.

### The TQT-Covid Strategy

The TQT-Covid-Strategy, part of a major project developed in two Brazilian municipalities, involved four research institutions, with collaboration and support from the Brazilian Ministry of Health (*Ministério da Saúde* – MS) and funding from an international agency (UNITAID). There was a local committee for the implementation, monitoring, and evaluation of the project, established in March 2022, with members of top-level management, boards of health care, Covid-19 response, surveillance and AHR, and researchers.

The TQT-Covid Strategy was developed based on literature review and used data from surveillance databases and protocols from different countries, validated by the World Health Organization, and national ones, validated by the MS^17^. It was structured in three components: expansion of accessibility to testing, monitoring of cases and surveillance strategies, and digital platform (TQT-Covid-System)^17,18^.

Standard operating procedures (SOPs) of the TQT-Covid-Strategy were made available to standardize the intervention; all health professionals who expressed interest had undergone training. Furthermore, during the implementation process, education actions for workers in the distance and face-to-face modalities were carried out, which will not be the object of this analysis.

Six health researchers (enablers) were present at each HU during the first seven days of the intervention, subsequently visiting the unit once a week, with continuous support to professionals via telephone and the WhatsApp application. There was also a professional hired by the TQT-Covid-Strategy to lead actions, support SOPs and expand testing.

### Process for Monitoring the Intervention and Data Analysis

For six months, the TQT-Covid-Strategy was monitored, with regular and systematic monitoring of the implementation of actions for each of its components (Chart 2). The actions and their results were also described, generating hypotheses about contextual factors that interfered in the implementation, as well as possible adjustments in its course. After the enablers’ visits, the observed results were taken to the TQT-Covid Implementation and Monitoring Committee, in order to discuss the implemented strategies.

**Chart 2:** Monitoring strategies of the TQT-Covid-Strategy intervention in an administrative health region of a municipality in Northeastern Brazil, 2022.

Strategy	Description of activities	Frequency
Presence of the enabler at HUs	Systematic monitoring of implementation; Support meetings and adjustments of flows with teams and management; Production of field diary; Production of report with activities developed in the HU, with record of the difficulties and problems faced.	Weekly
Observation script	Observation scripts of the units biweekly filled out by health professionals of the project, seeking to understand difficulties related to the testing flow in the units.	Biweekly
Monitoring workshops	Face-to-face workshops mediated by enablers and researchers with representatives of all HUs (workers and management) to understand problems in the implementation and support coping with it; Questionnaire on the implementation of the TQT-Covid-Strategy and the SWOT matrix.	Bimonthly
Indicator dashboard	Monitoring a dashboard of indicators produced by the TQT-System.	Weekly

HU: health unit; SWOT: strengths, weaknesses, opportunities and threats.

Source: prepared based on the intervention plan^17^.

Three face-to-face monitoring workshops were held, in which data from the implementation up to that moment were presented, as well as planning and elaboration of an action plan to address the gaps. This plan was monitored by the enablers and discussed again in subsequent workshops. In addition to sharing the experience and challenges of possible adjustments in the process, the workshops also sought to strengthen the support and increase the team's confidence in the intervention.

Through field diaries, weekly reports of the enablers, and reports on the workshops, matrices were created considering the activities planned in each of the three components of the intervention. The analysis was carried out by extracting excerpts from data sources, aiming to produce evidence on the extent the actions were being carried out. Subsequently, two external researchers analyzed the actions reported each month, classifying the situation for each of the components as adequate (A), when equal to or greater than 66%; partially adequate (P), when greater than 33% and lower than 66%; and inadequate (I), when lower than 33%. In this stage, there was the observation of what was previously established by the action plan and by the protocol of the TQT-Covid-Strategy^17,18^ (Chart 3). Each researcher classified the situation separately, with high agreement, and divergent items were analyzed again seeking to achieve consensus to finish the classification process.

**Chart 3:** Synthesis of the data produced by monitoring of the TQT-Covid-Strategy in the Family Health Units (FHUs 1 to 12), Health Centers (UBSs 1 to 5), and in an administrative health region of a municipality in Northeastern Brazil, 2022–2023.

	Expanded accessibility to testing				Monitoring of cases and surveillance strategies				Use of digital platform				
	Communication, risk communication, and health education in the territories, social equipment and dissemination in the community	Communication, risk communication, and health education in health units	Continuous testing	Testing for symptomatic individuals and contacts (symptomatic and asymptomatic individuals)	Active search for new cases (territory)	Active search for contacts (territory)	Telemonitoring of positive cases	Contact tracing	Notifications of cases	Registration of testing	Test results	Telemonitoring	Contact tracing
	Status (A, P, I)	Status (A, P, I)	Status (A, P, I)	Status (A, P, I)	Status (A, P, I)	Status (A, P, I)	Status (A, P, I)	Status (A, P, I)	Status (A, P, I))	Status (A, P, I)	Status (A, P, I)	Status (A, P, I)	Status (A, P, I)
FHU 1	A	A	A	A	I	I	P	P	A	A	A	I	I
FHU 2	P	A	A	A	I	I	I	I	A	A	A	I	I
FHU 3	A	A	A	A	I	I	P	I	A	A	A	I	I
FHU 4	A	A	A	A	A	I	I	I	A	A	A	I	I
FHU 5	A	P	A	A	I	I	P	P	A	A	A	I	I
FHU 6	A	A	A	A	P	I	I	I	A	A	A	I	I
FHU 7	A	A	A	A	P	I	P	I	A	A	A	I	I
FHU 8	P	A	A	A	I	I	P	P	A	A	A	I	I
FHU 9	A	A	A	A	I	I	I	I	A	A	A	I	I
FHU 10	A	A	A	A	I	I	I	NC	A	A	A	I	I
FHU 11	P	A	A	A	P	I	I	I	A	A	A	I	I
FHU 12	I	A	A	A	I	I	I	I	A	A	A	I	I
UBS 1	P	A	P	A	I	I	P	I	A	A	A	I	I
UBS 2	I	A	A	A	I	I	P	P	A	A	A	I	A
UBS 3	I	P	A	A	I	I	P	P	A	A	A	I	I
UBS 4	A	A	P	A	I	I	I	I	A	A	A	I	I
UBS 5	I	A	P	A	I	I	P	P	A	A	A	I	I

A: adequate; P: partially adequate; I: inadequate; NC: no cases.

Moreover, for the analysis of the component "expansion of accessibility to testing," notifications of cases recorded by the eSUS Notifica [eSUS Notifies] System, of the Ministry of Health, and the TQT-System during the intervention (July 2022 to April 2023) and the total of cases (suspected and confirmed) notified in the same months a year before the TQT-Covid-Strategy (July 2021 to April 2022) were compared (Table).

**Table.** Total and percentage of notifications recorded by the eSUS Notifica System of the Ministry of Health and the TQT-System in an administrative health region of a municipality in Northeastern Brazil, from July 2021 to April 2022 and from July 2022 to April 2023.

	Municipality (eSUS Notifica/MS)	AHR (eSUS Notifica/MS)	% AHR (eSUS Notifica/MS)	AHR (TQT-System)	% AHR/TQT-System in relation to HD/eSUS Notifica
Total cases (suspected and confirmed) (Jul/2022–Apr/2023)	61,225	9,424	15.4	6,838	72.5
Total confirmed cases notified (Jul/2022–Apr/2023)	13,871	1,739	12.5	–	–
Total cases (suspected and confirmed) notified in the same months (Jul/2021–Apr/2022), one year before the TQT-Strategy	157,296	15,368	9.8	–	–

AHR: Administrative Health Region; MS: Ministry of Health; HD: Health District.

Finally, we sought to infer in which aspects the monitoring was able to produce adjustments throughout the intervention, in addition to subsidizing the subsequent evaluation of the degree of implementation. Data were analyzed and validated by two researchers.

The study was approved by the Research Ethics Committee of the Instituto de Saúde Coletiva da Universidade Federal da Bahia, under CAAE Opinion No. 53844121.4.1001.5030. For ethical reasons of confidentiality, the name of the municipality was not disclosed.

## RESULTS

According to the monitoring analysis, the implementation of the TQT-Covid-Strategy differed between the 17 HUs (12 FHUs and five UBSs) in several elements of the three components of the intervention (Chart 3). It should be noted that testing was expanded in the AHR territory and was qualified in all 17 HUs, with provision of infrastructure (material, inputs, protocols, and SOPs), training of workers, monitoring, and support of professionals specialized in the area of public health (enablers).

### Expansion of Accessibility to Testing

The following strategies were proposed: communication, risk communication, and health education on Covid-19 prevention, testing, and operation of services and articulation with the community, carried out by community health agents (CHAs) and/or other professionals in the territory (distribution of pamphlets and posters in households, commercial establishments, schools, and others, advertising in sound trucks, news websites*,* and local television program). Teams used social media (WhatsApp, Instagram, and Facebook) in addition to the TQT-Covid-System itself. In the HUs, actions were carried out in waiting rooms, during consultations, including handing out the "Covid Kit," containing personal protective equipment and guidance to patients who tested positive.

Regarding the communication activities within the HUs, overall, they were adequate. However, in the activities in the territory, we observed variations in the two HU models, being adequate in eight FHUs and inadequate in three UBSs (Chart 3).

As for data on notifications, we found increased testing after the TQT-Covid-Strategy. Considering the total number of cases in the eSUS Notifica system for the municipality, during the intervention period (n = 61,225), 15.4% were registered in the analyzed AHR (Table). In comparison with the period from July 2021 to April 2022, the same period a year prior to the intervention, the percentage of notifications in the AHR increased from 9.8% to 15.4%, even with a better epidemiological scenario as of the second half of 2022 (Table).

Continuous testing throughout the period was considered adequate, with only three UBSs partially adequate. Testing for symptomatic individuals and contacts (symptomatic or asymptomatic) was deemed adequate. It is worth noting the difficulty recording contact tracing due to the patient's frequent refusal to provide their names and telephone numbers, which was attributed, among other factors, to the presence of drug trafficking in territories of great socioeconomic vulnerability. The demand for home testing and the printing of the test result was low, but these services were available.

### Monitoring of Cases and Surveillance Strategies

The TQT-Covid- Strategy prioritized the active search for new cases in the territories by CHAs or other professionals, referring identified cases for testing. This criterion was considered inadequate in most HUs (n = 12). Contacts should be contacted by telephone and, in case of no response, active search would be carried out. The latter was inadequate in all HUs. In the UBSs, the absence of CHAs was pointed out as a justification, while in the FHUs the number of these professionals was insufficient.

Regarding the telemonitoring of cases, by using the TQT-Covid system, it is noteworthy that all HUs received two tablets and had at least one computer. Nevertheless, frequent Internet instabilities were recorded in all HUs. In the UBSs, the majority were partially adequate (n = 4) and, in the FHUs, the inadequate result (n = 7) predominated (Chart 3). This classification was based on qualitative monitoring records, according to which telemonitoring was sometimes carried out by professionals using their own telephone devices, without registration on the platform.

The reported problems of the TQT-Covid-System for telemonitoring were duplication of records, indication of patients who had already been discharged, and difficulty opening several tabs simultaneously. In months with the highest number of cases (October and November 2022), professionals reported prioritizing testing. Other situations consisted in users without access to the Internet, who did not answer telephone calls, besides the lack of SIM chip to make calls.

Regarding contact tracing, the result varied from partially adequate to inadequate in the HUs. Professionals reported that these activities were not routinely performed, or the absence of positive cases, in addition to the users’ refusal to identify contacts.

### Uses of the Platform

Regarding the use of the platform for case notification, we observed that, of the total number of cases registered in the eSUS Notifica system of the Ministry of Health in the AHR (n = 9,424), during the intervention period, 72.5% (n = 6,838) were notified in the TQT-Covid-System (Table). Monitoring data showed inadequate use of the platform in relation to telemonitoring and contact tracing, and adequate result for the other analyzed items, which reflected difficulty in incorporating surveillance-related elements in the PHC work process (Chart 3).

Some difficulties and contextual aspects of the HUs may have influenced the low incorporation of the TQT-Covid-System by professionals, such as telemonitoring predominantly carried out by the nurse and/or dentist and, in most units, with the use of professionals’ own telephone devices, as well as technical issues of the platform, Internet instability, and professionals’ difficulty in using the platform. In months with the highest number of occurrences, there was delay in the notifications of suspected and confirmed cases in the TQT-Covid-System, with losses of these data.

## DISCUSSION

The monitoring process of the TQT-Covid-Strategy, based on the stage of implementation and progress^19,20^, enabled the systematization of the steps and instruments used, contributed to detect differences between the initial proposal of the intervention and the way it was developed and operationalized, allowing researchers to understand the main barriers toward the desired goals and changes as well as possible adjustments.

The results varied concerning UBSs and FHUs — FHUs accounted for the best results in the analyzed criteria. The time of six months for implementation and monitoring was marked by large oscillations in the number of Covid-19 cases in the city, with months without records, alternating with two months with significant increase. This situation interfered both in the development of the intervention and in the monitoring. In the periods without notification, the professionals focused on other activities of the work process, while, in the months of increased cases and work overload for the team, they reported not having time to carry them out, besides increase in professionals’ illnesses and leaves of absence.

Contextual variations, such as location, access, physical infrastructure, users’ flow, professional staffing, previous experience with testing, and health units created in the pandemic, were also highlighted. Better performance of the work process of the teams working in FHUs, compared to those working in UBSs, was pointed out, regarding the attributes and actions expected for PHC^23^, considering the differences such as better physical structure, higher workload of professionals, and the presence of CHAs in the family health strategy (FHS).

The role of CHAs was highlighted, especially in poorer countries, with shortage of health professionals, either for maintaining essential health services or facing the pandemic, with community education, active search, patient follow-up, and contact tracing. CHAs were also considered reliable sources of information^24^. To the everyday work, other responsibilities and actions related to the pandemic were added, resulting in fatigue, lack of motivation, and cases of psychic illness, factors also reported in other studies^25,26^. Policies on financial incentive, training, valorization, and more professional recognition can increase the involvement and maintenance of the actions of these workers^27^.

The TQT-Covid-Strategy sought to contemplate these incentives; however, the action of CHAs in the territories was compromised by the greater involvement of these professionals in activities within the HUs — such as user embracement. The presence of urban violence was highlighted as an aggravating factor.

The monitoring process did not contribute to modifying some problem situations, such as the non-feeding of the TQT-Covid-System for surveillance actions or not using platform resources, as expected. CHAs began to act in the units’ welcoming and reception, with fewer actions in the territories; in addition, the teams reported work overload, having professionals who were on leave due to health conditions and structural problems with the Internet.

The teams presented differences in reconciling the chronological time of the intervention with the appropriation of the proposal. There were differences regarding the "use value" of the involved innovations and changes and the engagement of professionals. The six-month period was not enough to incorporate all technological tools into the team's work process. We observed that the testing and notification interface was more used, at the expense of the surveillance-related interfaces.

It should be noted that the system was developed amid the pandemic to enable rapid and effective response, while facing the serious health crisis and its developments, with continuous maintenance. Thus, strategies for qualifying surveillance practices in PHC should be continued and implemented in a shared way with professionals in their everyday routine, with the inclusion and improvement of information and communications technology (ICT) tools to meet local needs, in addition to ongoing health education actions and other mechanisms to encourage good practices^2,28^.

Difficulties with the implementation of information technologies are very present in the reality of the Brazilian Unified Health System. Conflicts arising from the change in the routine of professionals and resistance to data recording partly justify it^29^. In this specific case, the situation was aggravated by the pandemic and collective illness. In periods of greater demand and increase of cases, the team was more susceptible to getting ill, frustration, difficulties in reconciling activities, and personal worries.

Authors of studies on the organization of primary health care in six countries in the Asia-Pacific region have shown that the majority used telehealth during the pandemic^29^. However, digital information systems did not properly work in the public sector, and the use of telecommunications in this context highlighted disparities and inequalities in the ability to manage information technologies and Internet access^29^, even in countries with levels of education and gross domestic product per capita higher than those in Brazil.

The partial or unsatisfactory results and contextual aspects found in the present study were consistent with those presented by a study on UBSs of three municipalities in the Northeast of Brazil, with emphasis on incipient surveillance actions, such as active search and risk communication in the territories. It was also pointed out that poor connectivity was a complicating factor for the using telemedicine, telemonitoring, and telescreening^30^. Finally, researchers showed that low PHC coverage and insufficient management professionals may have compromised the expansion of surveillance actions^30^.

Experiences in African and Asian countries, such as Ethiopia, China, and India, have shown that the local PHC-focused organization has also played a vital role in responding to the pandemic in middle- and low-income countries, focusing on mobilization to provide resources, inputs, and health professionals, case control, and risk communication^25,26^.

The best results in communication strategies, risk communication, and health education in the FHUs reinforce the importance of this model. Community support, especially in territories of great social vulnerability, health education in households and the media, including social media and instant messaging applications, has helped to increase public health awareness, demonstrating the potential of local governments to respond to public health emergencies and other disasters^24–26^.

## CONCLUSIONS

The analysis of the monitoring of the TQT-Covid-Strategy showed that systematic monitoring was essential for implementing the intervention, although it was not enough to promote all necessary adjustments in a timely manner and incorporate all components, especially those related to surveillance and the use of the digital platform. The most adequate results were found for the component concerning the expansion of accessibility to testing. This study will subsidize future evaluations to identify possible explanations and generate recommendations for improving new interventions.

We observed that the time (six months) was not enough for the complete implementation of the digital platform within the pandemic scenario and for the professionals’ use of all its resources. This, in addition to the gaps in health surveillance practices of PHC teams, reinforce that qualification strategies must be continued and promoted in a structural way so that this level of care is better prepared for their routine actions and adaptations in the face of new crises. As a challenge, we emphasize that initiatives such as these have their funding terminated after the pandemic period. To seek sustainability, a project planning and implementation committee was created with the participation of managers from different levels of municipal management.

Boosting the institutionalization of monitoring is an important instrument for the implementation and improvement of health interventions, particularly when integrated with planning and training evaluation, as in the analyzed case. In addition to an action protocol articulated at all care levels — and in the specific case —, it is recommended to systematize a PHC preparation plan, with emphasis on the use of digital platforms, considering the structure of the services and the ability to respond to them.

## Data Availability

The research data not included in the article are unavailable.
