# Supplementary material for: Analysis of the monitoring of the expansion of Covid-19 testing and surveillance in a municipality
Source: Rev Saude Publica. 2026 May 1;60(Suppl 1):e9s. doi: 10.11606/s1518-8787.2026060006945 (PMC13138129; doi:10.11606/s1518-8787.2026060006945)
Supplement: Translation

# Translation: Análise do monitoramento da expansão da testagem e vigilância da Covid-19 em um município

**Authors:** Alcione Brasileiro Oliveira, Ana Maria Freire de Souza Lima, Thais Regis Aranha Rossi, Gerluce Alves Pontes da Silva, Sisse Figueiredo de Santana, Trícia Silva Santos, Izabel Cristina Neves Ramos, Sintique Priscila Alves Lopes, Nathalia Almeida Suzart, Laio Magno, Inês Dourado, Lígia Maria Vieira da Silva

**Affiliations:** I Universidade Federal da Bahia. Instituto de Saúde Coletiva. Salvador, BA, Brasil; II Universidade Federal da Bahia. Faculdade de Odontologia. Departamento de Odontologia Social e Pediátrica. Salvador, BA, Brasil; III Universidade do Estado da Bahia. Departamento de Ciências da Vida. Salvador, BA, Brasil; IV Secretaria Municipal de Saúde de Camaçari. Camaçari, BA, Brasil; V Fundação Oswaldo Cruz. Instituto Gonçalo Moniz. Salvador, Bahia, Brasil

**Keywords:** Monitoramento em Saúde, COVID-19, Atenção Primária à Saúde, Tecnologia de Saúde Digital, Teste para COVID-19

## Abstract

**OBJETIVO:**

Analisar o monitoramento de uma intervenção para expansão de testagem, isolamento, quarentena e telemonitoramento (Estratégia-TQT-Covid) em uma região administrativa de saúde de um município do Nordeste brasileiro.

**MÉTODOS:**

Estudo avaliativo, cujo objeto de análise foram os dados produzidos no monitoramento de uma intervenção em saúde (Estratégia-TQT-Covid), durante seis meses, em 17 unidades de saúde, sendo 12 unidades de saúde da família e cinco unidades básicas de saúde. Foram analisadas matrizes de monitoramento construídas por meio de relatórios de campo, oficinas com profissionais e gestores e atividades de educação permanente. O monitoramento deu-se nos três componentes da intervenção Estratégia-TQT-Covid: ampliação da acessibilidade à testagem, acompanhamento de casos e estratégias de vigilância, e plataforma digital. Consideraram-se as ações em cada componente como adequadas (A), parcialmente adequadas (P) e inadequadas (I), em relação às atividades previstas no plano de ação e no protocolo da Estratégia-TQT-Covid.

**RESULTADOS:**

O componente da ampliação da acessibilidade à testagem foi considerado adequado, já o acompanhamento de casos e estratégias de vigilância apresentou resultados parcialmente adequados ou inadequados em muitas unidades. No componente plataforma digital, houve desempenho predominantemente adequado em relação ao registro e ao acesso aos resultados dos testes e notificação de casos. Contudo, o uso de outros recursos relacionados à vigilância, como rastreamento de contactantes, foi inadequado.

**CONCLUSÃO:**

O estímulo à institucionalização do monitoramento pode se constituir importante instrumento para implantação e aperfeiçoamento das intervenções sanitárias. A presença regular de profissionais facilitadoras e de um protocolo amplamente divulgado, além dos agentes comunitários de saúde, potencializou a intervenção. Contudo, os resultados parcialmente adequados ou inadequados reforçaram a importância da qualificação do processo de trabalho na atenção primária à saúde no que tange às ações de vigilância e ao uso de tecnologias da informação e da comunicação.

## INTRODUÇÃO

A emergência sanitária decretada pela Organização Mundial da Saúde, em fevereiro de 2020, seguida pela confirmação da pandemia de Covid-19 ocasionaram mais de 770 milhões de casos e quase 7 milhões de mortes acumuladas até 2023^1^. A situação levou os países a adotarem diversas medidas, sendo as principais a testagem, a identificação dos casos e seu isolamento, o rastreamento de contatos e sua quarentena, componentes centrais da vigilância epidemiológica^2–4^, além da reorganização da assistência, gerando consequências danosas, principalmente naqueles com maior desigualdade social^2,4^.

A atenção primária à saúde (APS) foi fundamental na ampliação da testagem e de outras estratégias de enfrentamento da Covid-19 em todo o mundo^5–7^. Países como Canadá, Reino Unido, Portugal, Espanha e Nova Zelândia destacaram-se também ao implementar rapidamente sistemas de acompanhamento e telemonitoramento, por meio da APS, para dar suporte aos pacientes e envolver-se no acompanhamento das doenças crônicas e agudas e nas ferramentas de telemedicina^8–11^.

No Brasil, apesar de as unidades básicas de saúde (UBS) e unidades de saúde da família (USFs) estarem presentes em todos os municípios, incluindo as áreas mais remotas do país, há poucas evidências sobre o monitoramento das intervenções propostas durante a pandemia, a incorporação de novos sistemas, recursos e ações de vigilância pelas equipes de APS, bem como a sustentabilidade dessa incorporação e seus aprendizados para cenários futuros^12–14^. Particularmente, sobre o uso de tecnologias de informação e comunicação, limitações para implementação são destacadas, sobretudo em locais de maior vulnerabilidade socioeconômica, com mais dificuldade de acesso à internet e baixo letramento digital^15,16^.

Em um município do Nordeste brasileiro, foi implementada uma estratégia para expansão de testagem, isolamento, quarentena e telemonitoramento (Estratégia-TQT-Covid) em 17 unidades de saúde (US) da APS numa região administrativa de saúde (RAS)^17,18^. Destaca-se que a implantação de nova intervenção precisa ser monitorada e avaliada por meio de uma abordagem formativa, pois assim é possível identificar, em estágio inicial, se os resultados desejados estão sendo alcançados, fazer correções, além de aumentar a probabilidade de obter resultados somativos confiáveis ao final do processo^19^.

O presente estudo analisou o monitoramento dessa intervenção, a Estratégia-TQT-Covid, durante seis meses, com observação semanal das US. Os resultados podem contribuir para o preparo para enfrentar pandemias futuras, por meio de experiências acumuladas, aprendizados e legados.

## MÉTODOS

Estudo avaliativo, cujo objeto de análise foram os dados produzidos no monitoramento da Estratégia-TQT-Covid, de julho de 2022 a fevereiro de 2023, em 17 US de uma RAS de um município da Região Nordeste do Brasil, sendo 12 USFs e cinco UBS. Esse monitoramento ocorreu durante seis meses, com observações conduzidas por uma equipe de pesquisadores e visitas semanais às US. O monitoramento foi compreendido como avaliação que se realiza ao longo do tempo^20^.

A abordagem adotada para a avaliação foi a formativa, com uso da proposta de classificação adaptada de Stetler et al.^19^ O monitoramento envolveu os estágios focados na implementação, ou seja, no processo, com acompanhamento no campo, conforme plano da intervenção, e no progresso, em direção às metas de implementação, com identificação de aspectos a serem abordados por meio de retorno (*feedback*) para as equipes e refinamento das estratégias^19^.

### Análise da Situação Inicial

O município onde se localiza a RAS analisada abriga aproximadamente 3 milhões de habitantes e 483 US, correspondendo à cobertura populacional da APS de 57,68%^20^. A RAS onde ocorreu a intervenção abrange população coberta de quase 400 mil habitantes, com 22 unidades de APS, sendo 10 UBS e 12 USFs^21^.

O desenho da Estratégia-TQT-Covid foi construído mediante análise situacional e diagnóstico das principais dificuldades para o enfrentamento da pandemia na referida RAS. Inicialmente, ocorreram quatro reuniões em dezembro de 2021, com 33 participantes (gerentes, profissionais de saúde e pesquisadores). A segunda fase dessa avaliação seguiu até março de 2022, com entrevistas semiestruturadas (profissionais e usuários) e grupos focais (profissionais)^22^. Os principais problemas identificados antes da intervenção foram relacionados à infraestrutura (materiais, insumos e recursos humanos), à organização e à gestão, além do processo de trabalho (Quadro 1).

**Quadro 1:** Principais problemas identificados na região administrativa de saúde de um município do Nordeste brasileiro antes da intervenção (2021–2022).

Infraestrutura, materiais, insumos
Infraestrutura inadequada
Ausência de dispositivos eletrônicos e rede de internet instável
Ausência de controle de estoque de EPI e insumos para testagem
Recursos humanos
Insuficiência de profissionais
Sobrecarga de trabalho e adoecimento dos trabalhadores
Organização e gestão
Testagem concentrada em poucas unidades de saúde
Despreparo e incertezas sobre protocolos e fluxos e falta de oferta de capacitações sobre Covid-19 por parte da gestão municipal
Ausência de canal de comunicação com os usuários
Preenchimento manual de fichas de notificações e problemas de notificação no sistema do Ministério da Saúde
Ausência de divulgação da ocorrência de casos de Covid-19 desagregados por área de abrangência da unidade de saúde
Monitoramento da situação de forma manual e/ou com uso de planilhas do *Microsoft Excel e Google*, com descontinuidades
Processo de trabalho
Baixa testagem com presença de filas e tempo de espera longo nas unidades de saúde
Ausência de estratégias de comunicação em saúde nos territórios sobre a Covid-19 (prevenção, sintomas, testagem e vacinação)
Ausência de identificação precoce de casos e contactantes
Baixa notificação de casos
Baixo índice de busca ativa (casos novos e contactantes)
Ausência ou baixo telemonitoramento
Dificuldades na realização, registro e sistematização do telemonitoramento — maior parte feita manualmente
Dificuldade de acesso aos laudos dos testes
Não rastreamento dos contactantes

EPI: equipamento de proteção individual.

Fonte: Relatório de Avaliação Formativa da Estratégia-TQT-Covid.

Das 22 US, cinco foram excluídas, pois eram geridas pelo setor privado ou estavam sem profissional da enfermagem e/ou com estrutura física muito limitada. Assim, a Estratégia-TQT-Covid envolveu 17 US, sendo 12 USFs e cinco UBS. Ressalta-se que, das 17 US, em sete já se realizavam testagens para Covid-19 prévia ou tinham alguma experiência nesse trabalho, as demais passaram a efetuar testes após a Estratégia-TQT-Covid; três USFs foram implantadas no período da pandemia.

### A Estratégia-TQT-Covid

A Estratégia-TQT-Covid, parte de um projeto amplo desenvolvido em dois municípios brasileiros, envolveu quatro instituições de pesquisa, com colaboração e apoio do Ministério da Saúde (MS) e financiamento de organismo internacional (UNITAID). Houve um comitê local de implementação, acompanhamento e avaliação do projeto, instituído em março de 2022, com membros da gestão do nível central, das diretorias de atenção à saúde, de resposta à Covid-19, de vigilância e da RAS e pesquisadores.

A Estratégia-TQT-Covid foi elaborada com apoio de uma revisão de literatura e utilizando dados de bases da vigilância e protocolos de distintos países, validados pela Organização Mundial da Saúde, e nacionais, pelo MS^17^, e estruturada em três componentes: ampliação da acessibilidade à testagem, acompanhamento de casos e estratégias de vigilância, e plataforma digital (Sistema-TQT-Covid)^17,18^.

Foram disponibilizados procedimentos operacionais-padrão (POPs) da Estratégia-TQT-Covid visando padronizar a intervenção; todos os profissionais de saúde que manifestaram interesse foram treinados. Ainda, durante o processo de implementação, houve ações de formação dos trabalhadores nas modalidades a distância e presencial, as quais não serão objetos desta análise.

Seis pesquisadoras sanitaristas (facilitadoras) estiveram em cada US durante os sete primeiros dias da intervenção, depois mantiveram a rotina de uma visita por semana, com apoio contínuo aos profissionais por telefone e aplicativo WhatsApp. Havia ainda uma profissional contratada pela Estratégia-TQT-Covid para liderar as ações, apoiar os POPs e ampliar a testagem.

### Processo de Monitoramento da Intervenção e Análise dos Dados

Durante seis meses, monitorou-se a Estratégia-TQT-Covid, com acompanhamento regular e sistemático de implementação das ações para cada um dos seus componentes (Quadro 2). Buscou-se ainda descrever as ações e os seus resultados, gerando hipóteses sobre fatores contextuais que interferiram na implementação, bem como possíveis ajustes no seu curso. Após as visitas das facilitadoras, os resultados observados foram levados para o comitê de implementação e acompanhamento do TQT-Covid, a fim de se discutir sobre as estratégias implementadas.

**Quadro 2:** Estratégias de monitoramento da intervenção Estratégia-TQT-Covid em uma região administrativa de saúde de um município do Nordeste brasileiro, 2022.

Estratégia	Descrição das atividades	Frequência
Presença da facilitadora na US	Acompanhamento sistemático da implementação; Reuniões de apoio e ajustes de fluxos com as equipes e gestão; Produção de diário de campo; Produção de relatório com atividades desenvolvidas nas US, com registro das dificuldades e dos problemas enfrentados.	Semanal
Roteiro de observação	Roteiros de observação das unidades preenchidos quinzenalmente por profissionais de saúde bolsistas do projeto, a fim de compreender dificuldades relacionadas ao fluxo de testagem nas unidades.	Quinzenal
Oficinas de monitoramento	Oficinas presenciais mediadas pelas facilitadoras e pesquisadores com representantes de todas as US (trabalhadores e gestão) para compreender os problemas na implantação e apoiar no seu enfrentamento; Questionário sobre a implantação da Estratégia-TQT-Covid e a matriz *SWOT*.	Bimensal
Painel de indicadores	Acompanhamento de painel de indicadores produzidos pelo Sistema TQT.	Semanal

US: unidade de saúde; *SWOT*: *strengths* (forças), *weaknesses* (fraquezas), *opportunities* (oportunidades) e *threats* (ameaças).

Fonte: Elaborado com base no plano da intervenção^17^.

Foram realizadas três oficinas presenciais de monitoramento, nas quais se apresentaram dados da implantação até aquele momento, bem como planejamento e elaboração de plano de ação para enfrentar as lacunas. Esse plano foi monitorado pelas facilitadoras e discutido novamente nas oficinas subsequentes. Além de compartilhar a experiência e os desafios de possíveis ajustes no processo, as oficinas também buscaram reforçar o apoio e aumentar a confiança da equipe em torno da intervenção.

Por meio dos diários de campo, relatórios semanais das facilitadoras e relatoria das oficinas, foram construídas matrizes considerando as atividades previstas em cada um dos três componentes da intervenção. A análise deu-se pela extração de trechos das fontes de dados, visando produzir evidências do quanto as ações estavam sendo realizadas. Depois disso, duas pesquisadoras externas analisaram as ações relatadas em cada mês, classificando a situação para cada um dos componentes em adequada (A), quando igual ou superior a 66%; parcialmente adequada (P), maior que 33% e inferior a 66%; e inadequada (I), abaixo de 33%. Essa etapa deu-se observando o que foi estabelecido previamente pelo plano de ação e pelo protocolo da Estratégia-TQT-Covid^17,18^ (Quadro 3). Cada pesquisadora fez a classificação separadamente, com elevada concordância, e aqueles itens divergentes foram objeto de nova análise em busca de consenso para finalizar a classificação.

**Quadro 3:** Síntese dos dados produzidos pelo monitoramento da Estratégia-TQT-Covid nas unidades de saúde da família (USFs 1 a 12), unidades básicas de saúde (UBS 1 a 5) e em uma região administrativa de saúde em um município do Nordeste brasileiro, 2022–2023.

	Ampliação da acessibilidade à testagem				Acompanhamento de casos e estratégias de vigilância				Uso de plataforma digital				
	Comunicação, comunicação de risco e educação em saúde nos territórios, equipamentos sociais e divulgação na comunidade	Comunicação, comunicação de risco e educação em saúde nas unidades de saúde	Testagem contínua	Testagem para sintomático e contactantes (sintomáticos e assintomáticos)	Busca ativa de casos novos (território)	Busca ativa de contactantes (território)	Telemonitoramento dos casos positivos	Rastreamento de contactantes	Notificações dos casos	Registro da testagem	Resultados de testes	Telemonitoramento	Rastreamento de contactantes
	Situação (A, P, I)	Situação (A, P, I)	Situação (A, P, I)	Situação (A, P, I)	Situação (A, P, I)	Situação (A, P, I)	Situação (A, P, I)	Situação (A, P, I)	Situação (A, P, I))	Situação (A, P, I)	Situação (A, P, I)	Situação (A, P, I)	Situação (A, P, I)
USF 1	A	A	A	A	I	I	P	P	A	A	A	I	I
USF 2	P	A	A	A	I	I	I	I	A	A	A	I	I
USF 3	A	A	A	A	I	I	P	I	A	A	A	I	I
USF 4	A	A	A	A	A	I	I	I	A	A	A	I	I
USF 5	A	P	A	A	I	I	P	P	A	A	A	I	I
USF 6	A	A	A	A	P	I	I	I	A	A	A	I	I
USF 7	A	A	A	A	P	I	P	I	A	A	A	I	I
USF 8	P	A	A	A	I	I	P	P	A	A	A	I	I
USF 9	A	A	A	A	I	I	I	I	A	A	A	I	I
USF 10	A	A	A	A	I	I	I	SC	A	A	A	I	I
USF 11	P	A	A	A	P	I	I	I	A	A	A	I	I
USF 12	I	A	A	A	I	I	I	I	A	A	A	I	I
UBS 1	P	A	P	A	I	I	P	I	A	A	A	I	I
UBS 2	I	A	A	A	I	I	P	P	A	A	A	I	A
UBS 3	I	P	A	A	I	I	P	P	A	A	A	I	I
UBS 4	A	A	P	A	I	I	I	I	A	A	A	I	I
UBS 5	I	A	P	A	I	I	P	P	A	A	A	I	I

A: adequado; P: parcialmente adequado; I: inadequado; SC: sem casos.

De forma complementar, para análise do componente de ampliação da acessibilidade à testagem, foram comparadas as notificações de casos registradas pelo sistema eSUS Notifica do Ministério da Saúde e no Sistema-TQT durante a intervenção (de julho de 2022 a abril de 2023) e o total de casos (suspeitos e confirmados) notificados nos mesmos meses de um ano antes da Estratégia-TQT-Covid (de julho de 2021 a abril de 2022) (Tabela).

**Tabela.** Total e percentual de notificações registradas pelo sistema eSUS Notifica do Ministério da Saúde e pelo Sistema-TQT em uma região administrativa de saúde de um município do Nordeste brasileiro, nos períodos de julho de 2021 a abril de 2022 e de julho de 2022 a abril de 2023.

	Município (eSUS Notifica/ MS)	RAS (eSUS Notifica/MS)	% RAS (eSUS Notifica/MS)	RAS (Sistema TQT)	% RAS/Sistema TQT em relação ao DS/eSUS Notifica
Total de casos (suspeitos e confirmados) jul/2022–abr/2023	61.225	9.424	15,4	6.838	72,5
Total de casos confirmados notificados jul/2022–abr/2023	13.871	1.739	12,5	-	-
Total de casos (suspeitos e confirmados) notificados nos mesmos meses jul/2021–abr/2022, um ano antes da Estratégia TQT	157.296	15.368	9,8	-	-

RAS: região administrativa de saúde; MS: Ministério da Saúde; DS: distrito sanitário.

Por fim, buscou-se inferir em quais aspectos o monitoramento foi capaz de produzir ajustes no curso da intervenção, além de subsidiar a posterior avaliação do grau de implantação. Os dados foram analisados e validados por duas pesquisadoras.

O estudo foi aprovado pelo Comitê de Ética em Pesquisa do Instituto de Saúde Coletiva da Universidade Federal da Bahia, sob parecer de número CAAE 53844121.4.1001.5030. Por razões éticas de confidencialidade, o nome do município não foi divulgado.

## RESULTADOS

A análise do monitoramento revelou diversidade na implementação da Estratégia-TQT-Covid entre as 17 US (12 USFs e cinco UBS) em vários elementos dos três componentes da intervenção (Quadro 3). Destaca-se que a testagem foi ampliada no território da RAS e qualificada em todas as 17 US, com disponibilização de infraestrutura (material, insumos, protocolos e POPs), formação de trabalhadores, acompanhamento e apoio de profissionais especialistas na área de saúde pública (facilitadoras).

### Ampliação da Acessibilidade à Testagem

Foram propostas estratégias de comunicação, comunicação de risco e educação em saúde sobre prevenção de Covid-19, testagem e funcionamento dos serviços e articulação com a comunidade, aplicadas pelos agentes comunitários de saúde (ACS) e/ou demais profissionais no território (distribuição de panfletos e cartazes em domicílios, estabelecimentos comerciais, escolas e outros dispositivos, divulgação em carro de som nas ruas, *site* jornalístico e programa televisivo local). As equipes utilizaram redes e mídias sociais (*WhatsApp, Instagram e Facebook*), além do próprio Sistema-TQT-Covid. Nas US, foram realizadas ações nas salas de espera, consultas, incluindo a entrega do "Kit Covid", contendo equipamento de proteção individual e orientações aos pacientes com testagem positiva.

Em relação às atividades de comunicação dentro das US, no geral, elas foram adequadas. Contudo, nas atividades no território, observaram-se variações nos dois modelos de US, sendo adequadas em oito USFs e inadequadas em três UBS (Quadro 3).

Os dados analisados sobre as notificações mostraram ampliação da testagem após a Estratégia-TQT-Covid. Considerando o total de casos constantes no eSUS Notifica para o município, no período da intervenção (n = 61.225), 15,4% foram registrados na RAS analisada (Tabela). Na comparação com o período de julho de 2021 a abril de 2022, o mesmo de um ano antes da intervenção, o percentual de notificações na RAS aumentou de 9,8% para 15,4%, mesmo com cenário epidemiológico melhor a partir do segundo semestre de 2022 (Tabela).

A testagem contínua durante todo o período foi considerada adequada, com apenas três UBS parcialmente adequadas. A testagem para sintomático e contactante (sintomático ou assintomático) foi considerada adequada. Destaca-se a dificuldade de registrar o rastreamento de contactantes pela recusa frequente do paciente de indicar nome e telefone, o que foi atribuído, dentre outros fatores, à presença do tráfico de drogas em territórios de alta vulnerabilidade socioeconômica. A demanda por testagem em domicílio e a impressão do resultado do teste foi baixa, porém eram serviços disponíveis.

### Acompanhamento de Casos e Estratégias de Vigilância

A Estratégia-TQT-Covid priorizou a busca ativa de casos novos nos territórios por ACS ou outros profissionais, com encaminhamento do caso identificado para testagem. Esse critério foi considerado inadequado na maioria das US (n = 12). Os contactantes deveriam ser contactados por telefone e, em caso de não resposta, haveria busca ativa. Esse último foi inadequado em todas as US. Como justificativa, nas UBS, foi apontada a ausência de ACS, e nas USFs, o número desses profissionais foi insuficiente.

Quanto ao telemonitoramento dos casos, com uso do Sistema-TQT-Covid, destaca-se que todas as US receberam dois *tablets* e tinham pelo menos um computador. Contudo, foram registradas instabilidades frequentes na rede de internet em todas as US. Nas UBS, a maioria foi parcialmente adequada (n = 4) e, nas USFs, predominou o resultado inadequado (n = 7) (Quadro 3). Essa classificação baseou-se nos registros qualitativos do monitoramento, os quais apontaram que o telemonitoramento, por vezes, foi feito pelos profissionais por meio dos próprios aparelhos telefônicos, sem o registro na plataforma.

Os problemas do Sistema-TQT-Covid para o telemonitoramento relatados foram duplicidade de cadastros, indicação de pacientes que já tinham recebido alta e dificuldade de abrir várias abas simultaneamente. Nos meses de maior número de casos (outubro e novembro de 2022), os profissionais argumentaram priorizar a testagem. Outras situações foram usuários sem acesso à internet, que não atendiam às chamadas telefônicas, além da falta de *chip* para efetuar ligações.

Quanto ao rastreamento de contactantes, o resultado variou de parcialmente adequado a inadequado nas US. Foram relatadas a não realização dessas atividades de forma rotineira, ou ausência de casos positivos, além da recusa dos usuários em apontar contactantes.

### Usos da Plataforma

Quanto ao uso da plataforma para notificação dos casos, observou-se que, do total de casos registrados no sistema eSUS Notifica do Ministério da Saúde na RAS (n = 9.424), no período da intervenção, 72,5% (n = 6.838) foram notificados no Sistema-TQT-Covid (Tabela). Os dados do monitoramento demonstraram uso inadequado da plataforma em relação ao telemonitoramento e ao rastreamento e resultado adequado para os demais itens analisados, o que refletiu dificuldade de incorporar elementos da vigilância no processo de trabalho da APS (Quadro 3).

Algumas dificuldades e aspectos contextuais das US relatadas podem ter influenciado a baixa incorporação do Sistema-TQT-Covid pelos profissionais, tais como telemonitoramento predominantemente pela enfermeira e/ou dentista e, na maioria das unidades, com uso de aparelho telefônico pessoal, bem como problemas técnicos na plataforma, instabilidade na conexão da internet e dificuldade de profissionais no manejo da plataforma. Nos meses de maior número de ocorrências, houve atraso nas notificações dos casos suspeitos e confirmados no Sistema-TQT-Covid, com perdas desses dados.

## DISCUSSÃO

O processo de monitoramento da Estratégia-TQT-Covid, com base no estágio de implementação e de progresso^19,20^, possibilitou sistematizar etapas e instrumentos utilizados, contribuiu para detectar diferenças entre a proposta inicial da intervenção e o modo como ela foi desenvolvida e operacionalizada, permitindo aos investigadores compreender as principais barreiras em direção aos objetivos e a mudanças desejadas, bem como possíveis ajustes.

Houve variação nos resultados de UBS e USFs — os melhores nos critérios analisados foram os das USFs. O tempo de seis meses para implantação e monitoramento foi marcado por grandes oscilações no número de casos de Covid-19 no município, com meses sem registros, alternando com dois meses com aumento significativo. Essa situação interferiu tanto no desenvolvimento da intervenção como no monitoramento. Nos períodos sem notificação, os profissionais voltavam-se para outras atividades do processo de trabalho, ao passo que, nos meses de aumento de casos e sobrecarga de trabalho para a equipe, relataram não conseguir tempo para efetuá-las, além de aumento do adoecimento e afastamento de profissionais.

Destacam-se também variações contextuais, como localização, acesso, infraestrutura física, fluxo de usuários, provimento profissional, experiência prévia de testagem e ainda unidades de saúde criadas na pandemia. Melhor desempenho do processo de trabalho das equipes de USF, comparadas às de UBS, foi apontado, no que tange a atributos e ações esperadas para a APS^23^, considerando as diferenças, como melhor estrutura física, maior carga horária de profissionais e a presença de ACS na ESF.

O papel dos ACS foi ressaltado, principalmente em países mais pobres, com escassez de profissionais de saúde, seja pela manutenção dos serviços essenciais de saúde, seja para enfrentar a pandemia, com educação comunitária, busca ativa, acompanhamento de pacientes e rastreamento de contactantes. Os ACS foram também considerados fontes confiáveis de informações^24^. Ao trabalho cotidiano, foram acrescidas outras responsabilidades e ações relacionadas à pandemia, resultando em cansaço, desmotivação e casos de adoecimento psíquico, fatores também relatados em outros trabalhos^25,26^. Políticas de incentivo financeiro, formação, valorização e mais reconhecimento profissional podem aumentar o envolvimento e a manutenção das ações desses trabalhadores^27^.

A Estratégia-TQT-Covid buscou contemplar esses incentivos, entretanto, a ação dos ACS nos territórios ficou comprometida pelo maior envolvimento desses profissionais em atividades nas US, como acolhimento de usuários. Destacou-se como fator agravante a presença da violência urbana.

O processo de monitoramento não contribuiu para modificar algumas situações-problema, a exemplo da não alimentação do Sistema-TQT-Covid para as ações de vigilância ou não uso de recursos da plataforma, conforme previsto. Os ACS passaram a atuar no acolhimento e na recepção das unidades, com quantidade menor de ações nos territórios, assim como as equipes relatavam sobrecarga de trabalho com profissionais afastados por condições de saúde e problemas estruturais da rede de internet.

As equipes apresentaram diferenças em conciliar o tempo cronológico da intervenção com a apropriação da proposta. Houve diferenças sobre o "valor de uso" das inovações e das mudanças envolvidas e no engajamento dos profissionais. O período de seis meses não foi suficiente para incorporar todas as ferramentas tecnológicas ao processo de trabalho das equipes. Observou-se que a interface de testagem e notificação foi mais utilizada, em detrimento das interfaces relativas à vigilância.

Ressalta-se que foi um sistema desenvolvido em plena pandemia para possibilitar resposta rápida e efetiva, em meio à grave crise sanitária e seus desdobramentos, com manutenção contínua. Assim, estratégias de qualificação das práticas de vigilância na APS precisam ser continuadas e implementadas de maneira compartilhada com os profissionais em seu cotidiano, com a inclusão e o aperfeiçoamento das ferramentas de tecnologia da informação e comunicação (TIC) para atender às necessidades locais, além de ações de educação permanente em saúde e outros mecanismos de incentivo a boas práticas^2,28^.

Dificuldades com a implementação de tecnologias de informação estão muito presentes na realidade do Sistema Único de Saúde. Conflitos decorrentes da mudança da rotina dos profissionais e resistência ao registro de dados justificam-nas, em parte^29^. No caso específico, a situação foi agravada pela pandemia e pelo adoecimento coletivo. Nos períodos de maior demanda e aumento de casos, a equipe esteve mais susceptível a adoecimentos, frustrações, dificuldades em conciliar as atividades e receios pessoais.

Investigações sobre a organização de cuidados primários em saúde em seis países da região Ásia-Pacífico mostraram que a maioria utilizou a telessaúde durante a pandemia^29^. Contudo, os sistemas de informação digital não funcionavam plenamente no setor público, e o uso das telecomunicações nesse contexto destacou disparidades e desigualdades na capacidade de manejar tecnologias de informação e no acesso à internet^29^, mesmo em países com níveis de escolaridade e produto interno bruto *per capita* mais altos que os do Brasil.

Os resultados parciais ou insatisfatórios e os aspectos contextuais encontrados no presente estudo coadunaram com aqueles apresentados por um estudo em UBS de três municípios do Nordeste brasileiro, com ênfase na incipiência nas ações de vigilância, como a busca ativa e a comunicação dos riscos nos territórios. Também foi apontado que a incipiente conectividade se apresentou como complicador para as ações de uso da telemedicina, do telemonitoramento e do telerrastreio^30^. E, por fim, evidenciaram que a baixa cobertura de APS e a insuficiência de profissionais na gestão podem ter comprometido a ampliação das ações de vigilância^30^.

Experiências em países da África e da Ásia, como Etiópia, China e Índia, demonstraram que a organização local centrada na APS também desempenhou papel vital na resposta à pandemia em países de médio e baixo rendimentos, com foco na mobilização para suprir recursos, insumos e profissionais de saúde, controle de casos e comunicação de riscos^25,26^.

Os melhores resultados nas estratégias de comunicação, comunicação de risco e educação em saúde nas USFs reforçam a importância desse modelo. O apoio comunitário, sobretudo em territórios de alta vulnerabilidade social, a educação em saúde nos domicílios e nos meios de comunicação, incluindo redes sociais e aplicativos de mensagens instantâneas, ajudaram a aumentar a consciência pública sanitária, demonstrando o potencial dos governos locais para responder a emergências de saúde pública e outras catástrofes^24–26^.

## CONCLUSÃO

A análise do monitoramento da Estratégia-TQT-Covid mostrou que o acompanhamento sistemático foi fundamental para implementar a intervenção, ainda que não tenha sido suficiente para promover todos os ajustes necessários em tempo oportuno, e incorporar todos os componentes, especialmente aqueles relacionados à vigilância e ao uso da plataforma digital. Os resultados mais adequados foram registrados no componente ampliação da acessibilidade à testagem. Este estudo subsidiará avaliações futuras para identificar possíveis explicações e gerar recomendações para melhoria de novas intervenções.

Observou-se que o tempo (seis meses) não foi suficiente para implementação completa da plataforma digital, no cenário da pandemia, bem como para uso de todos os seus recursos pelos profissionais. Isso somado às lacunas nas práticas de vigilância em saúde das equipes de APS reforçam que as estratégias de qualificação precisam ser continuadas e fomentadas de maneira estruturante para que esse nível de atenção esteja mais bem preparado para suas ações de rotina e adaptações diante de novas crises. Ressalta-se como desafio que iniciativas como essas encerram seu período de financiamento após o período da pandemia. Para buscar sustentabilidade, foi criado um comitê de planejamento e implementação do projeto com participação de gestores de distintos níveis da gestão municipal.

O estímulo à institucionalização do monitoramento constitui-se importante instrumento para implantação e aperfeiçoamento das intervenções sanitárias, particularmente quando integrado ao planejamento e à avaliação formativa, como no caso analisado. Além de um protocolo de ação articulado em todos os níveis de atenção — e no caso específico —, recomenda-se a sistematização de um plano de preparação da APS, com ênfase no uso de plataformas digitais, considerando a estrutura dos serviços e a capacidade de responder a eles.

## Data Availability

Os dados da pesquisa não inclusos no artigo não estão disponíveis.
